# Tick attachment cement – reviewing the mysteries of a biological skin plug system

**DOI:** 10.1111/brv.12384

**Published:** 2017-11-08

**Authors:** Johannes Suppan, Benedikt Engel, Martina Marchetti‐Deschmann, Sylvia Nürnberger

**Affiliations:** ^1^ Department of Trauma Surgery Austrian Cluster for Tissue Regeneration, Medical University of Vienna, Währinger Gürtel 18‐20 A‐1090 Vienna Austria; ^2^ Institute of Chemical Technologies and Analytics, TU Wien, Getreidemarkt 9/164 A‐1060 Vienna Austria

**Keywords:** ticks, Ixodidae, blood feeding, cement plug, artificial feeding, bioadhesives, salivary glands

## Abstract

The majority of ticks in the family Ixodidae secrete a substance anchoring their mouthparts to the host skin. This substance is termed cement. It has adhesive properties and seals the lesion during feeding. The particular chemical composition and the curing process of the cement are unclear. This review summarizes the literature, starting with a historical overview, briefly introducing the different hypotheses on the origin of the adhesive and how the tick salivary glands have been identified as its source. Details on the sequence of cement deposition, the curing process and detachment are provided. Other possible functions of the cement, such as protection from the host immune system and antimicrobial properties, are presented. Histochemical and ultrastructural data of the intracellular granules in the salivary gland cells, as well as the secreted cement, suggest that proteins constitute the main material, with biochemical data revealing glycine to be the dominant amino acid. Applied methods and their restrictions are discussed. Tick cement is compared with adhesives of other animals such as barnacles, mussels and sea urchins. Finally, we address the potential of tick cement for the field of biomaterial research and in particular for medical applications in future.

## I. INTRODUCTION

Numerous animals produce chemical adhesives. Not surprisingly, the different lifestyles of these animals have led to the evolution of adhesives with different chemical compositions and properties. Remarkably, these gluing substances can solidify rapidly and bond to diverse surfaces, even under water (Flammang & Santos, 2015). Additionally, they are biodegradable after different periods of time and, due to their biological origin, in most cases they are also biocompatible.

Due to these properties, biological glues have significant potential for special applications, particularly in medicine but also for industry (Stewart, Ransom & Hlady, 2011). Nevertheless, only a few adhesives have been studied in detail, such as the byssus threads of mussels (Qin *et al*., 2016), barnacle cement (Kamino *et al*., 2000), and the glue used by Sandcastle worms (Zhao *et al*., 2005). Most of these aquatic organisms use glues based on modified amino acid side chains, especially phosphorylated serines and hydroxylated tyrosines [e.g. 3,4‐dihydroxyphenylalanine (DOPA)] (Stewart *et al*., 2011). However, for the majority of other biological adhesives the composition and bonding mechanisms are largely unknown.

One example is a substance called ‘cement’, which is produced by ticks to anchor their mouthparts firmly into the skin tissue of their hosts during a blood meal. The natural function of this cement suggests that this material might have adhesive properties that could potentially be useful in the development of medical tissue glues or sealants. Here, we summarize current knowledge about the morphology, composition and mechanisms of attachment cement in ticks.

Ticks (Ixodida) are subdivided into three families: Nuttalliellidae (one species), Argasidae (193 sp.) and Ixodidae (702 sp.) (Guglielmone *et al*., 2010). During their life cycle, they develop over successive egg, larva and nymph (up to eight in Argasidae) stages to adults (Oliver, 1989; Apanaskevich & Oliver, 2014). All species are temporary ectoparasites feeding obligatorily on the blood of vertebrates (Coons & Alberti, 1999). During feeding, the blood is ingested from a cavity in the host tissue (feeding pool or lesion) and the animals alternate between blood uptake and the injection of saliva into it (Sonenshine, 2005).

The production of attachment cement is known in the family Ixodidae, whereas it seems to be absent or at least uncommon in Argasidae (Binnington & Kemp, 1980; Kemp, Stone & Binnington, 1982). There are no reports of cement production in *Nuttalliella namaqua* Bedford, 1931, the single species in the family Nuttalliellidae (Mans *et al*., 2012). The presence of cement seems to be related to different tick feeding habits; nymphs and females of *Nuttalliella namaqua* (Mans *et al*., 2011) and most life stages in the Argasidae complete their blood meal within minutes to hours (Oliver, 1989; Apanaskevich & Oliver, 2014) and therefore may not need additional anchorage. Furthermore, the Argasidae enter the host skin deeply (Sauer *et al*., 1995) with well‐developed mouthparts (Binnington & Kemp, 1980) and usually become active when their host animals are resting or sleeping in their nests or burrows (Oliver, 1989), perhaps making cement production unnecessary. Exceptions might be found in larval stages of some genera in Argasidae and the larvae of *Nuttalliella namaqua* (Mans *et al*., 2012)*,* which take blood meals over several days (Oliver, 1989; Mans *et al*., 2012; Apanaskevich & Oliver, 2014). However, to date, cement has only been reported for feeding larvae of *Argas pusillus* Kohls, 1950 (Stiller & Ranchitham, 1975), although this observation is not undisputed (Kemp *et al*., 1982). It is likely that larval stages of the Argasidae do not need cement because the majority of this family is nidicolous (i.e. live in nests or burrows of their hosts) and therefore easily relocate a host after incidential detachment.

In contrast to Argasidae and Nuttalliellidae, long feeding periods are typical for all blood‐feeding life stages in Ixodidae, usually taking several days or even more than a week (Oliver, 1989). Accordingly, cement is thought to be produced in all genera of this family with the only known exceptions being some species in the genus *Ixodes* (Kemp *et al*., 1982).

## II. HISTORICAL PERSPECTIVE ON CEMENT RESEARCH

The attachment cement of ticks was first described in the early 20th century, when it was termed ‘cement‐like substance’ (Cowdry & Danks, 1933) or ‘homogeneous eosinophilic mass’ (i.e. with a strong affinity to acid dyes like eosin) (Hoeppli & Feng, 1931). Such structures around the mouthparts were histologically reported in skin biopsies from attached ixodid ticks on a variety of host animals (e.g. mice, hamsters, guinea pigs, sheep, dogs) (Hoeppli & Feng, 1931; Foggie, 1959; Gregson, 1960; Saito & Ohara, 1961; Theis & Budwiser, 1974).

Studies comparing the attachment of different tick species revealed differences in the position and shape of cement formations, as well as the insertion depth of the mouthparts (Saito & Ohara, 1961; Moorhouse, 1969). In agreement with these findings, genus‐specific patterns of attachment were distinguished (e.g. ‘*Ixodes* type’ and ‘*Haemaphysalis* type’) (Moorhouse, 1969). Among species of the genus *Ixodes* attachment types were found to be so variable that they were further subdivided (e.g. ‘*persulcatus* type’, ‘*japonensis* type’ ‘*Ixodes* group 1’, ‘*Ixodes* group 2’). In species of this genus cement could be totally absent, restricted to the area around the mouthparts within the host tissue, or additionally deposited on the skin surface (Saito & Ohara, 1961; Moorhouse, 1969).

For descriptive purposes, new terminologies (Table 1) were introduced for specific portions of cement (e.g. ‘perirostral cement’; Saito & Ohara, 1961). Some of these terms are related to the position of a cement portion only, while others also describe a particular composition (e.g. lipoprotein for the ‘internum’; Moorhouse, 1969).

**Table 1:** Terminology for cement portions

Terminology	Cement portion	References
Core cement	First portion of cement deposited at the attachment site, ‘internum’ (see below)	Kemp *et al*. (1982)
Cortical cement	Portion of cement laid down around the core cement and invading between the layers of the stratum corneum, ‘cortex’ (see below)	Binnington & Kemp (1980)
Internum	One of the two main portions of cement which consists of lipoprotein	Moorhouse & Tatchell (1966)
Cortex	One of the two main portions of cement which consists of carbohydrate‐containing protein (in *R. microplus*)	Moorhouse & Tatchell (1966)
External cement	Cement portion deposited on the skin surface, comprising a conical part (or cone) surrounding the mouthparts of the tick and lateral wing‐like extensions	Chinery (1973)
Internal cement	Cement portion below the skin surface, further separated into an inner and outer zone The compact inner zone consists of a tapering tube and the outer zone forms strands which are intermeshed with the fibrils of the surrounding dermal tissue	Chinery (1973)
Primary cement	Cement portion laid down on the first day of attachment, the cortex and internum can be clearly distinguished; the cement around the mouthparts	Binnington & Kemp (1980) and Moorhouse & Tatchell (1966)
Secondary cement	Portion of cement laid down approximately 24 h before the final engorgement process; cortical material is added to the base of the original cone extending it more deeply into the feeding cavity; the cement which is secreted into the feeding lesion	Binnington & Kemp (1980) and Moorhouse & Tatchell (1966)
Perirostral cement	Portion of cement laid down around the hypostome and cheliceral shafts, linked with the connective fibres in the corium of the host skin.	Saito & Ohara (1961)
Cover cement	Portion of cement which surrounds the greater part of the perirostral cement and covers the skin surface at the site of infestation	Saito & Ohara (1961)

Despite the frequent observation of cement, opinions on its nature and origin differed widely with some suggesting that cement was a host product. For instance, ‘conical or sleeve‐like papillae’ were described macroscopically on preserved tick‐infested skin samples (*Dermacentor* sp., *Amblyomma* sp., *Rhipicephalus* sp.). These papillae were interpreted as outgrowths of the skin, probably resulting from attempts by the host to engulf the parasite (Snodgrass, 1948). Based on histological observations from other tick species [*Ixodes ricinus* (Linnaeus, 1758), *I. trianguliceps* Birula, 1895], it was also suggested that the collagen in the host skin aggregates around the tick's mouthparts to form a tight‐fitting sheath, allowing firm attachment of the tick (Arthur, 1970, 1973; Whitwell, 1978).

Among those in favour of a tick‐derived origin, several sources were discussed: regurgitated (back flowing) material from the gut, the coxal glands and the salivary glands. The idea that cement was regurgitated fluid from the gut was rejected due to the absence of material with similar histological staining properties in the digestive tract (Cowdry & Danks, 1933). Coxal glands also were ruled out (Hoeppli & Feng, 1931; Cowdry & Danks, 1933), because secretory activity could not be observed, macroscopically nor in histological sections (Hoeppli & Feng, 1931). Indeed, coxal glands are characteristic of non‐cement‐producing Argasidae but are absent in cement‐producing Ixodidae (Chinery, 1973). Salivary glands were suggested to be the most likely source of the cement (Hoeppli & Feng, 1931; Gregson, 1960; Moorhouse & Tatchell, 1966; Moorhouse, 1969; Chinery, 1973): they become highly active during feeding and contain large amounts of secretory products (Cowdry & Danks, 1933), portions of the salivary glands react to histological staining in a similar way to the cement (Moorhouse & Tatchell, 1966; Moorhouse, 1969; Chinery, 1973), and a rapidly hardening fluid is secreted between the mouthparts of *Dermacentor andersoni* Stiles, 1908 following attachment to mouse ears (Gregson, 1960). Some evidence did challenge a salivary gland origin, for example intradermal injections of whole gland extracts did not produce cement‐like structures in laboratory animals (Foggie, 1959). However, an increasing number of reports on similar reactions of cement and intracellular granules of certain salivary gland cells to histochemical dyes (Coons & Roshdy, 1973; Gill & Walker, 1988), the absence of such cells and their products in ticks that do not secrete cement (Roshdy & Coons, 1975; Binnington & Kemp, 1980) and findings that the gland cells and the cement contain similar immunoreactive polypeptides (Jaworski *et al*., 1992) consolidated the salivary glands as the production site of cement.

Until the early 1990s, information on cement was primarily based on histological descriptions of host skin biopsies. Improved technologies paved the way for the modern molecular biological techniques that now dominate research on salivary glands and their products. Cement proteins with antigenic properties (e.g. 64P) were identified, steering research towards anti‐tick vaccines (Mulenga *et al*., 1999; Bishop *et al*., 2002; Trimnell, Hails, & Nuttall, 2002; Trimnell *et al*., 2005; Zhou *et al*., 2006). The establishment of complementary DNA (cDNA) libraries allowed the comparison of putative cement proteins in different tick species showing the variety in salivary gland composition (Maruyama *et al*., 2010). Nevertheless it became clear that the sheer quantity of proteins and other products of the salivary glands (Alarcon‐Chaidez, 2014) make assignment to particular functions such as cement formation difficult. To address this problem, temporary expression patterns of the salivary glands were analysed (Radulovic *et al*., 2014; Kim *et al*., 2016; Bullard, Williams & Karim, 2016), showing which salivary products were produced at certain points during feeding. These subsets of proteins were then tested for their functions by specific inactivation in living ticks and verification in feeding trials (Kim, Curran & Mulenga, 2014). However, some proteins of salivary glands have very similar nucleotide sequences, hampering efforts at specific silencing (Bullard *et al*., 2016).

**Figure 1:**
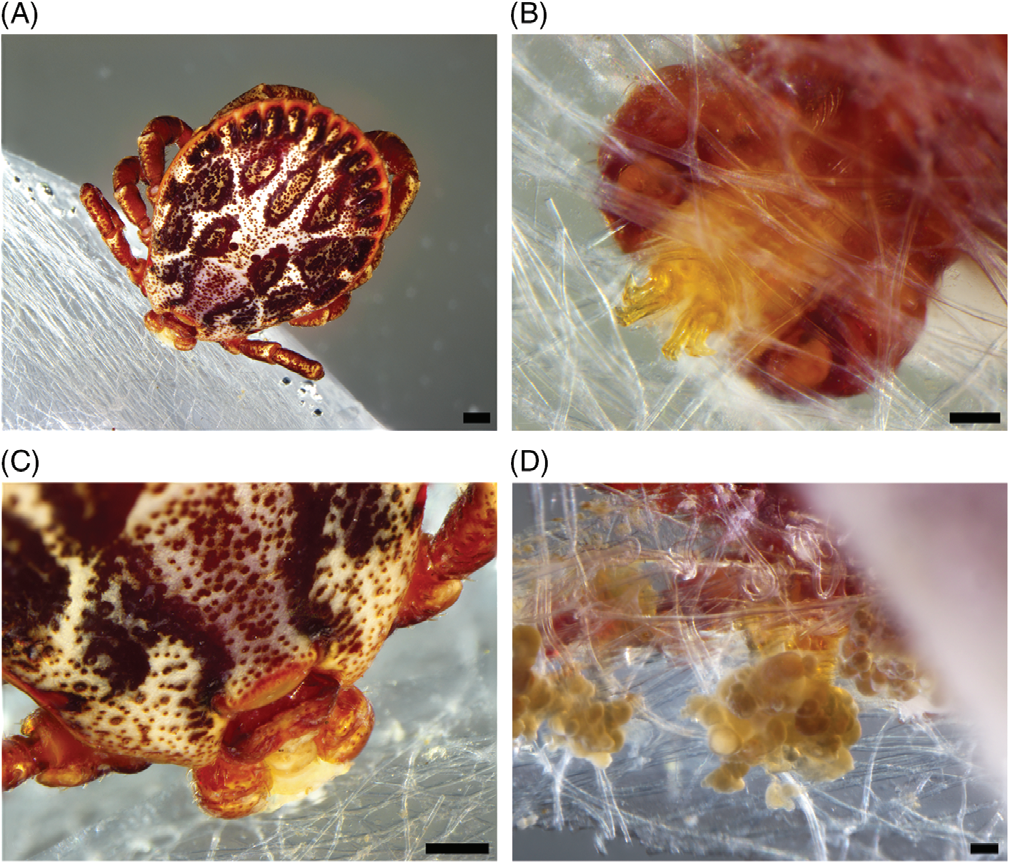
Artificial feeding of ticks. (A) Dermacentor marginatus (Sulzer, 1776), male attached to a silicone membrane. (B) Chelicerae visible after cutting through the membrane. (C) Cement deposition between the palps at the upper side, and (D) at the underside of the membrane. Scale bars: A, C = 300 µm; B, D = 100 µm.

Another approach in cement analysis was to obtain material free of host skin tissue (Bullard *et al*., 2016) by *in vitro* feeding of ticks on artificial membranes (Fig. 1). Feeding on animal‐derived and artificial membranes is well established for the non‐cement‐producing Argasidae but attempts to transfer these systems to Ixodidae initially failed because of more sophisticated requirements for mimicking specific host cues for acceptance and attachment to such membranes. The long feeding periods of Ixodidae present an additional challenge, demanding several blood changes per day and careful monitoring and chemical additives to prevent microbial growth (Kuhnert, 1996). Silicone membranes have been used successfully to feed adults of *Dermacentor nuttalli* Olenev, 1928 and *Hyalomma excavatum* Koch, 1844 (formerly *H*. *anatolicum excavatum*) (Habedank, Hiepe & Montag, 1994) and all life stages of *Amblyomma hebraeum* Koch, 1844 (Kuhnert, Diehl & Guerin, 1995) and *Ixodes ricinus* (Kroeber & Guerin, 2007). Even though the technique must be adapted and modified for use with each tick species according to the length of the mouthparts (Kroeber & Guerin, 2007), examples of successful feeding of other species such as *Rhipicephalus sanguineus* Latreille, 1806, *Hyalomma dromedarii* Koch, 1844 and *H. anatolicum* Koch, 1844 (formerly *H. anatolicum anatolicum*) (Tajeri & Razmi, 2011; Fourie *et al*., 2013) suggest this is a useful approach for cement research (Fig. 1). Compared to *in vivo* feeding, this method allows monitoring the progress of cement deposition and contamination‐free collection of samples (Bullard *et al*., 2016).

## III. BIOLOGICAL SIGNIFICANCE AND FUNCTIONS OF THE CEMENT

**Figure 2:**
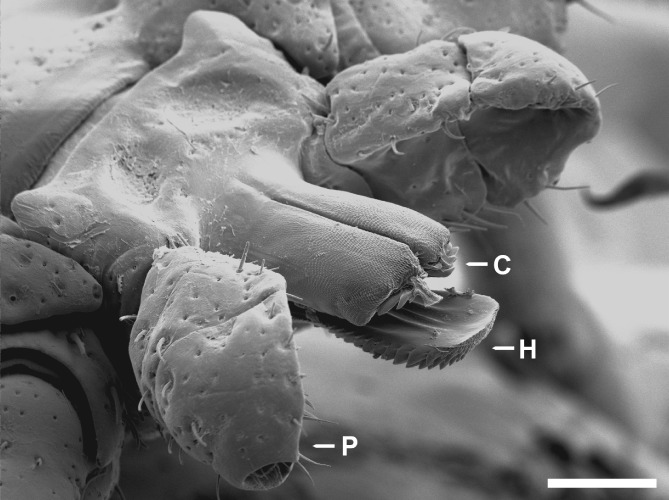
Mouthparts of a female Dermacentor marginatus. Between two palps (P) there are paired chelicerae (C) and the hypostome (H). The chelicerae are extendable and cut into the host tissue during attachment. The hypostome acts dorsally as channel for saliva and blood, ventrally there are rows of teeth anchoring the tick to the cement and host tissue. The palps remain at the skin surface and fulfil sensory functions. Scale bar = 200 µm.

Attachment cement is a rapidly hardening substance produced by the salivary glands of Ixodidae in preparation for and during feeding. Before cement secretion is finished, attached ticks can easily be pulled out of the skin (Sonenshine, 2005), but in manual tests considerable force is required to detach a tick after cement formation is completed (Gregson, 1960; Moorhouse & Tatchell, 1966). This demonstrates the anchoring function of the cement by strengthening the attachment of the ticks' mouthparts (Fig. 2) to the host. An effective form of attachment is essential for Ixodidae due to long feeding periods, during which the tick is at risk from host movement and scratching.

Besides its anchorage function, the deposited cement also fills any gaps between the inserted mouthparts and the host skin (Fig. 1). This sealing prevents loss of fluids and increases the effectiveness of the muscular pharynx, which is responsible for blood uptake (Saito & Ohara, 1961; Coons & Alberti, 1999). Intense bleeding at the feeding sites of *Amblyomma americanum* (Linnaeus, 1758) after silencing presumed cement compounds (Kim *et al*., 2014) corroborates this assumption.

In addition, it was reported that tick cement might have antimicrobial properties (Alekseev *et al*., 1995), but it remains unknown if these effects are derived from cement compounds themselves or from other salivary compounds in the cone (Francischetti *et al*., 2009). A related function might be the exclusion of bacteria on the host skin surface from the feeding pool (P.M. Guerin, personal communication). The suggested functions of cement are summarized in Table 2.

**Table 2:** Suggested functions of tick cement cones during feeding

Function	References
Firm attachment to the host	Coons & Alberti (1999), Sauer *et al*. (1995) and Theis & Budwiser (1974)
Sealing of the feeding lesion, to prevent the loss of fluids, enhance blood uptake and prevent the entry of air	Coons & Alberti (1999) and Saito & Ohara (1961)
Completing the channel formed by the mouthparts and directing the saliva more effectively into the host tissue	Arthur (1970); Tatchell (1969)
Obtaining blood (and other host fluids) from skin layers located much deeper than the distal ends of the mouthparts	Balashov (1984)
Blocking the expansion of the feeding pool into the tissues lateral to the mouthparts to maintain effective attachment	Arthur (1970); Tatchell (1969)
Preventing host immune molecules from coming into contact with the tick mouthparts	Binnington & Kemp (1980), Bishop *et al*. (2002) and Mulenga *et al*. (1999)
Confining the feeding pool within the host to prevent bacterial contamination from the host skin surface	P. M. Guerin (personal communication)
Antimicrobial activity	Alekseev *et al*. (1995) and Francischetti *et al*. (2009)

Cement production probably provides other advantages. The adhesive substance allows them to attach to a host with minimal invasion of the mouthparts into the host tissue, which are kept in place by the superficial cement cone. It has been noted for *Haemaphysalis* sp., which have this type of attachment, that the host skin is less damaged and that the healing process progresses more rapidly than for *Ixodes* species (Saito, Ohara & Unagami, 1960). Species of the latter genus in this study produced no, or only small cement deposits at the host skin surface (Saito & Ohara, 1961). It might be the case that host animals are more likely to develop defence mechanisms against tick species causing more damage. This might represent a selective advantage for tick species which invest into deposits of cement on the host skin surface instead of long mouthparts for secure attachment.

It is well known that ticks are able to reattach following involuntary detachment from hosts (Gregson, 1960; Jaworski *et al*., 1992; Wang, Henbest & Nuttall, 1999; Weiss & Kaufman, 2004). Cement production allows superficial attachment of ticks to the host and it might be speculated that this is advantageous during grooming by the host to remove the parasite. Gregson (1960) reported that the cement cone is usually pulled away with the mouthparts, as observed for *Dermacentor andersoni*. The cement might therefore function to protect the tick mouthparts during forceful detachment.

## IV. THE SEQUENCE OF CEMENT DEPOSITION

Throughout this review, we will use the terms ‘core’ and ‘cortical cement’ introduced by Kemp *et al*. (1982) (see Table 1).

Cement secretion by ixodid ticks starts within 5–30 min of the insertion of the mouthparts into the host skin (Kemp *et al*., 1982). The first portion of secretion is the core cement, which hardens rapidly. About 24 h later a second portion, the cortical cement is secreted, which solidifies more slowly (Kemp *et al*., 1982).

In *Rhipicephalus* (formerly *Boophilus*) *microplus* (Canestrini, 1888) a distinct core and cortical cement can be observed about 24 h after attachment. With continued feeding and growth of the cement the two portions become difficult to distinguish by histochemistry (Moorhouse & Tatchell, 1966). One possible explanation might be that the cortical cement is able to enter the core cement through small perforations (Kemp *et al*., 1982). Meanwhile, the cortical cement also spreads over and penetrates into the host skin close to the feeding site (Kemp *et al*., 1982) where it fills gaps, resulting in firm adhesion of the feeding tick. In general, cement deposition ceases after a few days of attachment (Kemp *et al*., 1982). However, some reports indicate that further cement can be deposited as secondary cement during the final stages of feeding (Moorhouse & Tatchell, 1966; Moorhouse, 1969).

In the three‐host tick (i.e. the host is left after the blood meal and each life stage has to seek a new host) *Haemaphysalis spinigera* Neumann, 1897, a species lacking secondary cement production, it was reported in females that cement production is almost complete after 36 h (Chinery, 1973). At feeding sites of female *Rhipicephalus sanguineus* no substantial increase in the amount of cement was observed after 24 h of attachment (Theis & Budwiser, 1974); however, secondary cement production was observed in another report on this species (Moorhouse, 1969).

Secondary cement is similar to cortical cement in its composition (Moorhouse & Tatchell, 1966) and adds material to the base of the original cone. As a result, the cement cone extends deeper into the feeding cavity and forms a flange (Binnington & Kemp, 1980). Some ticks (e.g. *Haemaphysalis* and *Dermacentor* sp.) have strong lateral cement flanges on the skin surface with little secondary cement production, while in others (e.g. *Rhipicephalus* spp.) there is an internal flange of secondary cement (Moorhouse, 1969).

For *Rhipicephalus microplus*, a one‐host tick (i.e. all parasitic stages feed on the same host individual) secondary cement production was observed on the third, 11th and 17th day for larvae, nymphs and females, respectively (Moorhouse & Tatchell, 1966), following initial attachment at the larval stage. It was suggested that these additional depositions of cement help the tick to remain securely attached following extension of the feeding cavity below the initial cone (Moorhouse & Tatchell, 1966), but they probably also protect the tick from detachment during the moulting process which also occurs on the host. Whereas moulting progresses uniformly all over the body of three‐host ticks, in nymphs of *R. microplus* this process begins earlier in the legs than in the mouthparts, which are still involved in feeding. Ticks of this life stage begin to become immobile on the 11th day following attachment (Jorgensen & Kemp, 1986), i.e. corresponding to the period of secondary cement production in nymphs (Moorhouse & Tatchell, 1966). However, no secondary cement deposition was found in histological studies of the three‐host ticks *Haemaphysalis spinigera*, *Hyalomma anatolicum* and *R. appendiculatus* Neumann, 1901 (Chinery, 1973; Walker & Fletcher, 1986; Gill & Walker, 1988).

## V. THREE SALIVARY GLAND CELLS AS ORIGIN OF THE CEMENT

**Figure 3:**
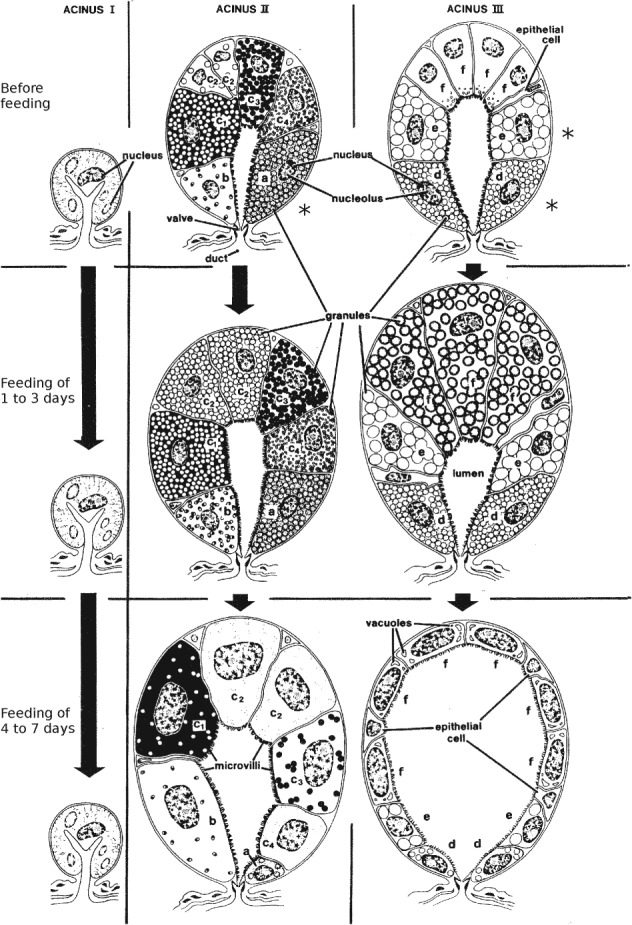
Acini and cell types of the salivary glands of the tick Rhipicephalus microplus (female) and changes that occur during feeding. Type I acini remain unchanged during feeding, whereas there are pronounced alterations in acini II and III. The position of a‐, d‐ and e‐cells involved in cement production are shown by asterisks. Image adapted from Kemp et al. (1982). (Reprinted with kind permission of Elsevier).

There is now general agreement that cement compounds originate from the tick paired salivary glands (Alarcon‐Chaidez, 2014; Sonenshine & Roe, 2014) which undergo remarkable structural changes to act both as a secretory organ and fluid‐transport system during feeding (Bowman, Ball & Sauer, 2008). These complex glands resemble clusters of grapes (Nicholson *et al*., 2009) with a single structural unit termed an acinus. The salivary glands consist of a large number of acini with three to four different types of secretory regions and a supporting duct system. In female *Rhipicephalus appendiculatus* each branch of the paired glands consists of about 1400 acini and there are about 1350 in males (Walker, Fletcher & Gill, 1985). In general, agranular (type I) and granular (types II, III and IV) acini can be distinguished. With the exception of type IV, each acinus type contains different groups of cells (Fig. 3). Cement seems to originate from the cell class a‐ of type II acini, and from d‐ and e‐cell classes in type III acini (Sauer *et al*., 1995; Coons & Alberti, 1999). Early observations of type IV acini were all from cement‐producing tick species (Fawcett, Binnington & Voigt, 1986), but there are no histochemical indications that they contribute material to the cement cone (Furquim, Bechara & Camargo Mathias, 2010). Indeed, type IV acini are also found in males of *Ixodes holocyclus* Neumann, 1899, a species which does not produce cement (Moorhouse, 1969) and in which males do not feed (Stone & Binnington, 1986).

Within their respective acini, a‐, d‐ and e‐cells are located close to the junction with the salivary duct system (Fig. 3) (Kemp *et al*., 1982). These cells contain secretory granules before attachment and the period of their secretion corresponds with that of cement deposition (Kemp *et al*., 1982; Gill & Walker, 1988). Similar staining of the secretory granules and the deposited cement in detailed histochemical studies of *Hyalomma anatolicum* (Gill & Walker, 1988) supports the hypothesis that a‐, d‐ and e‐cells are the origin of cement. Jaworski *et al*. (1992) localized a polypeptide in the d‐ and e‐cells in type III acini of the salivary glands of female *Dermacentor variabilis* (Say, 1821) by immuno‐staining which also appeared to be part of the cement (type II acini were not examined). The labelling in d‐cells was particularly evident and appeared stronger in cells from the unfed stage than in fed ticks. Interestingly, this particular polypeptide also seems to be present in the salivary glands of *Amblyomma americanum* and *Rhipicephalus sanguineus* ( Jaworski *et al*., 1992).

Additionally, *Rhipicephalus* immuno‐dominant molecule 36 (RIM 36 protein) cloned from a cDNA was found to be present in the salivary glands as well as the cement cones of *Rhipicephalus appendiculatus*. It was highly abundant in e‐cells, present in d‐cells and absent in type II acini (Bishop *et al*., 2002).

It has been observed in *Rhipicephalus appendiculatus* that d‐ and e‐cells of females are depleted of granules early in the feeding process and diminish in size afterwards, indicating that granule secretion contributes to cement deposition. By contrast, these cells remain synthetically active in males and lose fewer granules. This could be explained by the longer on‐host periods in males and repeated cement production (Fawcett *et al*., 1986). During copulation, male ixodid ticks change position to feed together with the female as a couple (Feldman‐Musham, 1986). Accordingly, cement might be needed more than once by the males (Binnington, 1978).

In a less‐common cell‐class classification, some studies (Chinery, 1973; Yanagawa *et al*., 1988) report a‐ (acinus II), c‐ and d‐ (both in acinus III in this classification) cells as involved in cement production. Differences in the cell types observed and in the nomenclature of salivary gland cells by different authors (Table 3) complicate the picture. Nevertheless, the similarities of these cells (Yanagawa *et al*., 1987) suggest that the same cell types were described.

**Table 3:** Differences in nomenclature for granular salivary gland cells. Cement precursor cells are highlighted in bold

Acinus	Most common cell type classification	Less‐common cell type classification

	(Binnington, 1978)	(Chinery, 1973; Yanagawa *et al*., 1987)
II	**a**	**a**
	b	b
	c (c1–c4)	—
III	**d**	**c**
	**e**	**d**
	f	e
IV	g	—

In comparison, salivary glands of non‐cement‐producing Argasidae are less complex with only one granular acinus type (Roshdy & Coons, 1975; Binnington & Kemp, 1980; Coons & Alberti, 1999). *Argas persicus* (Oken, 1818) (Argasidae) contains two cell types, with the described i‐cells resembling b‐cells of type II acini in *Haemaphysalis spinigera* (Ixodidae) (Chinery, 1974). Three cell types (a‐, b‐, and c‐) were described in the granular acinus of *Argas arboreus* Kaiser, Hoogstraal and Kohls, 1964 (Roshdy & Coons, 1975) and *Ornithodoros moubata* (Murray, 1877) (El Shoura, 1985). However, their granules all lack the microstructure described for Ixodidae (see Section VI), supporting a role of those structures in Ixodidae in the production of cement (Roshdy & Coons, 1975; El Shoura, 1985). No information could be found regarding the morphology and number of acini in *Nuttalliella namaqua* salivary glands, the non‐cement‐producing single species of the family Nuttalliellidae (Mans *et al*., 2011).

## VI. HISTOCHEMISTRY AND ELECTRON MICROSCOPY OF THE INTRACELLULAR GRANULES

Granules produced in the salivary gland cells have been subjected to histochemical testing in several species. Correlative histochemical data are available on gland cell granules (Table 4) and the cement material (Table 5) for four different tick species (*Haemaphysalis spinigera*, *Hyalomma anatolicum*, *Rhipicephalus appendiculatus* and *R. microplus*). These are the only species in which histochemical data been published for both gland cell granules and cement material.

**Table 4:** Summary of histochemical results on granules in salivary gland cells of different tick species. Histochemical methods applied in different studies may differ slightly, meaning that comparability is restricted to some extent (for details, see original publications). Symbols: +, positive reaction; −, negative reaction; 0, not tested; x, tested, but no data. Note, a‐, c‐ and d‐cells in Haemaphysalis are the same cell types as the a‐, d‐ and e‐cells in the other listed species. DOPA, 3,4‐dihydrophenylalanine

Components and enzymatic activities tested in the salivary granules	Tick species											
	*Haemaphysalis spinigera*			*Hyalomma* * anatolicum*			*Rhipicephalus* *appendiculatus*			*Rhipicephalus* * microplus*		

	(Chinery, 1973)			(Gill & Walker, 1988)			(Walker *et al*., 1985)			(Binnington, 1978)		
	Cell types											
	a	c	d	a	d	e	a	d	e	a	d	e
Carbohydrate	−	−	−	−	−	−	−	−	−	−	−	−
Acid mucopolysaccharide	−	−	−	0	0	0	0	0	0	0	0	0
Non‐specific protein	+	+	+	+	+	+	+	+	+	+	+	+
Lipoprotein	0	0	0	0	0	0	0	0	0	+	+	+
Tryptophan	+	+	+	x	+	+	−	+	+	+	+	+
Tyrosine	+	+	+	+	+	+	+	+	+	−	+	+
Arginine	−	−	−	0	0	0	0	0	0	0	0	0
Amino group	−	−	−	0	0	0	0	0	0	0	0	0
Sulphydryl group	+	+	−	+	+	+	+	+	+	+	+	+
Disulphide group	+	+	−	+	+	+	0	0	0	−	−	−
Phenolic group	0	0	0	+	+	+	+	+	+	+	+	+
Lipid	−	−	−	+	+	+	+	+	+	+	+	+
Nucleic acids	−	−	−	−	−	−	0	0	0	0	0	0
Esterase	0	0	0	−	−	−	−	−	−	0	0	0
Aminopeptidase	0	0	0	−	−	−	x	+	x	0	0	0
Sulphatase	0	0	0	−	−	+	0	0	0	0	0	0
β‐glucuronidase	0	0	0	−	−	−	0	0	0	0	0	0
Acid phosphatase	0	0	0	+	+	−	+	−	−	0	0	0
Alkaline phosphatase	0	0	0	−	−	−	−	−	−	0	0	0
Adenosine triphosphatase	0	0	0	−	−	−	−	−	−	0	0	0
Catechol oxidase/DOPA oxidase	0	0	0	−	−	−	+	+	+	0	0	0
Cytochrome oxidase	0	0	0	−	−	−	0	0	0	0	0	0
Monoamine oxidase	0	0	0	−	−	−	−	−	−	0	0	0
Peroxidase	0	0	0	0	0	0	−	+	−	0	0	0
NADPH diaphorase	0	0	0	−	−	−	0	0	0	0	0	0

**Table 5:** Summary of histochemical results from cement cones of Haemaphysalis spingera, Hyalomma anatolicum, Rhipicephalus appendiculatus and Rhipicephalus microplus. Histochemical methods in the different studies may differ slightly (for details, see original publications). Symbols: +, positive reaction; −, negative reaction; +/−, faint reaction; 0, not tested; (+), indirectly proven. DOPA, 3,4‐dihydrophenylalanine

	Tick species			
	*Haemaphysalis spinigera*	*Hyalomma anatolicum*	*Rhipicephalus appendiculatus*	*Rhipicephalus microplus*
Components and enzymatic activities tested in cement	(Chinery, 1973)	(Gill & Walker, 1988)	(Walker & Fletcher, 1986)	(Moorhouse & Tatchell, 1966)
Carbohydrate	−	−	−	+
Acid mucopolysaccharide	−	0	0	0
Non‐specific protein	+	+	+	0
Carbohydrate‐containing protein	0	0	0	+
Basic protein	0	0	0	+
Tryptophan	+	+	+	+
Tyrosine	+	+	+	0
Arginine	−	0	0	−
Amino group	−	0	0	0
Sulphydryl group	+	+/−	+	+
Disulphide group	+/−	+/−	0	(+)
Phenolic group	0	0	+	+
Lipid	+/−	+	+	+
Nucleic acid	−	0	0	0
Esterase	0	+/−	0	0
Aminopeptidase	0	+	+	0
Sulphatase	0	−	0	0
β‐glucuronidase	0	−	0	0
Acid phosphatase	0	+/−	0	0
Alkaline phosphatase	0	−	0	0
Adenosine triphosphatase	0	−	0	0
Catechol oxidase/DOPA oxidase	0	−	−	0
Cytochrome oxidase	0	+/−	0	0
Monoamine oxidase	0	−	0	0
NADPH diaphorase	0	−	0	0

All gland cells and cements stain for protein as well as the amino acids tyrosine and tryptophan. Lipids or lipoprotein were found in the granules of all cement‐producing salivary gland cells, except for *Haemaphysalis spinigera*, which only gave faint positive reactions in the cement and a negative reaction in the gland cells (Chinery, 1973).

Based on the Periodic acid Schiff reaction (PAS), carbohydrates are present in the cement of some, but not all species (Moorhouse, 1969). However, the PAS does not allow further differentiation so it is unknown whether these carbohydrates are free or bound to proteins, or if they are mono‐ or polysaccharides. For *Dermacentor variabilis*, which also contains carbohydrate in the cement (Moorhouse, 1969), faint or moderate reactions in granules of unfed males were reported (Coons & Roshdy, 1973). Surprisingly, there was no positive reaction for carbohydrate in the granules of *R. microplus* (Binnington, 1978), even though the (cortical) cement of this species stains intensively with the PAS (Moorhouse & Tatchell, 1966).

In the three species tested for phenolic groups, the granules in the gland cells tested positive, in accordance with the cement staining for two of these species (Binnington, 1978; Walker *et al*., 1985; Gill & Walker, 1988). Phenols can be converted into quinones, therefore their presence might indicate that a tanning process that crosslinks proteins is involved in cement formation (see Section IX).

Enzymatic activities were tested in the granules for two of the four species (*H. anatolicum* and *R. appendiculatus*). Activity of acid phosphatase, catechol oxidase, sulphatase and aminopeptidase observed in one or both species might be associated with modifications of secretory products in the granules. It is also possible that these enzymes are involved in other functions of tick saliva, such as the formation of the feeding pool. Granules in both species reacted positively for acid phosphatase. In *H. anatolicum*, granules of a‐ and d‐cells reacted strongly; in *R. appendiculatus*, a strong reaction was only found in a‐cell granules (Walker *et al*., 1985; Gill & Walker, 1988).

*R. appendiculatus* granules in all three cell types tested positive for catechol oxidases. In addition, d‐cell granules show moderate positive reactions for aminopeptidase and peroxidase (Walker *et al*., 1985). Sulphatases were tested for in *H. anatolicum* only and detected in e‐cell granules which gave a weak reaction (Gill & Walker, 1988). Catechol oxidase activity might be involved in quinone formation, again suggesting a possible role for tanning in cement formation. However, this enzymatic activity might also be associated with blood feeding; a salivary peroxidase of the mosquito *Anopheles albimanus* Wiedemann, 1820 also shows catechol oxidase activity and is known to cause vasodilation (Ribeiro & Valenzuela, 1999; Ribeiro, Mans & Arca, 2010). Similarly, the observed weak peroxidase activity might arise from a glutathione peroxidase, which is known from tick saliva and probably acts as an anti‐inflammatory substance at the feeding site (Tirloni *et al*., 2015). Observations of moderate aminopeptidase activity may correspond with the presence of dipeptidases in the saliva. Dipeptidases are thought to inhibit pain responses of the host following tick attachment by destruction of bradykinin (Tirloni *et al*., 2015).

Transmission electron microscopy (TEM) observations on the granules in salivary gland cells have revealed typical microstructural details, best described in *Rhipicephalus appendiculatus*. The ultrastructure of the granules in this species is very similar to that in comparable cell types in other tick species (Yanagawa *et al*., 1987).

The a‐cells of acinus II contain membrane‐bound secretory granules of up to 3 µm with round or oval subunits of 0.5 µm, surrounded by electron‐dense rod‐like structures. The granules are embedded in a substance which is usually more electron‐dense than the granules themselves; however, in some observations the granules appeared more dense than the surrounding substance (Walker *et al*., 1985; Fawcett *et al*., 1986). In females, the a‐cells remain conspicuous until the end of feeding, although they do become smaller. In males, they remain relatively unchanged during feeding (Walker *et al*., 1985).

The d‐cells of acinus III have granules very similar in appearance to those of a‐cells. The granules are 3–4 µm in diameter, often with very dense subunits of 0.4–0.6 µm in an electron‐lucent substance, often also containing irregular strands. These might represent a different granule component or another form of the contents from the dense subunits (Fawcett, Buscher & Doxsey, 1982). Similar to a‐cells, the density of the granule components may vary. It is not clear whether this is due to maturation processes or the result of differing fixation and dehydration steps in the various studies. High numbers of granules are found in unfed ticks, which are greatly reduced at the onset of feeding, when cement deposition starts. The observation of early granule stages within the Golgi apparatus suggests that d‐cells continue to produce their secretory product for some days, before the cells diminish in size in females. In males, a reduction in granules is less obvious, with TEM revealing that the cells continue to be synthetically active, which seems to be related to the capacity for repeated cycles of cement production and feeding in order to mate with several females on their host (Fawcett *et al*., 1986).

The cytoplasm of e‐cells in acinus III contains the largest granules seen in the glands of unfed ticks (4–6 µm). They are less electron‐dense than the granules in a‐ and d‐cells and spherical or ovoid in shape (Fawcett, Doxsey & Buscher, 1981). At lower magnifications, the granules appear amorphous but higher resolution reveals close packaging of subunits (50–100 nm) within a denser substance, giving the granules a fine reticular pattern. Additionally, small numbers of electron‐dense inclusions (100–500 nm) are randomly distributed throughout these granules. Similar to d‐cells, the e‐cells in males are active for longer than in females; in the latter the cells regress after exocytosis of their secretory products during attachment (Fawcett *et al*., 1981, 1986).

Electron microscopy is very limited regarding the identification of substances, unless special staining methods are applied. However, the granular ultrastructure of a‐ and d‐cells indicates that similar secretory products could be stored within these cells, whereas the content of e‐cell granules is obviously different in both structure and density. These electron microscopic observations correspond with histochemical data on unfed males of *D. variabilis* (Coons & Roshdy, 1973) where a‐ and d‐cell granules showed a moderate PAS reaction (for carbohydrate), stained blue with aniline blue black (ABB, for proteins) and violet with Toluidine Blue (for metachromasia). Granules of e‐cells, by contrast, showed only a faint reaction with PAS, blue‐to‐green staining with ABB and orthochromasia with Toluidine Blue. It would be beneficial to perform further studies comparing the histology and ultrastructure of the granules in a larger number of tick species at different points during feeding.

## VII. THE STRUCTURE OF CEMENT

Macroscopically, tick cement is usually white or pale brown in colour (Kemp *et al*., 1982). The material has been described as cell free (Foggie, 1959; Moorhouse & Tatchell, 1966) and homogeneous except for some striae, giving the cement a lamellate appearance which presumably results from its discontinuous deposition (Cowdry & Danks, 1933; Arthur, 1953; Moorhouse & Tatchell, 1966; Theis & Budwiser, 1974). In the cement of *Rhipicephalus appendiculatus*, Cowdry & Danks (1933) observed fine granular layers alternating with homogeneous layers. At the margin of the cement, strand‐like extensions are seen to intermesh with the surrounding dermis (Chinery, 1973; Walker & Fletcher, 1986) and imprints of the tick mouthparts are frequently described from sectioned material (Saito & Ohara, 1961; Chinery, 1973; Theis & Budwiser, 1974).

**Figure 4:**
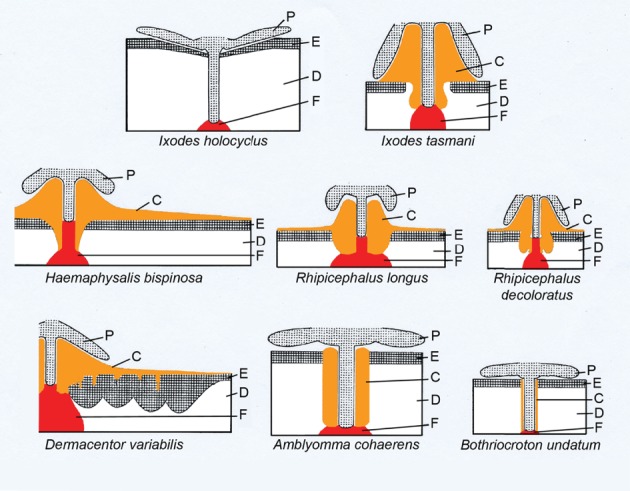
Examples of types of attachment by female ixodid ticks. The cement can form superficial cones of different sizes, with pronounced lateral flanges, or support the inserted mouthparts. Additional cement can infiltrate the host epidermis around the feeding site in Dermacentor females. Ixodes holocyclus is given for comparison, as this species does not produce cement. Symbols: C, cement; D, dermis; E, epidermis; F, feeding pool; P, palps. Image adapted from Moorhouse (1969). (Reprinted with kind permission of Akadémiai Kiadó).

Amongst ixodid ticks, cement cones differ in size and shape (Moorhouse, 1969). In general, two types of cement deposition can be distinguished (Fig. 4): (*i*) some species restrict the area of cement deposition close to the introduced mouthparts in the skin (e.g. ticks with long mouthparts found in the genera *Amblyomma* and *Bothriocroton*); (*ii*) many species secrete additional deposits of various dimensions onto the host surface. These surface deposits can form high cones at the feeding site (e.g. *Ixodes tasmani* Neumann, 1899) or form lateral flat flanges of various dimensions (e.g. *Haemaphysalis bispinosa* Neumann, 1897, *Dermacentor variabilis*, *Rhipicephalus longus* Neumann, 1907). High cones are known from species with long mouthparts, and lower cones from those with short mouthparts. In some species (e.g. *Rhipicephalus* spp.) which produce less‐extensive lateral flanges on the skin surface, internal flanges of secondary cement can be found instead (Moorhouse, 1969). Cement aggregations on the skin allow ticks with small mouthparts a secure attachment, but some tick species with longer mouthparts also use superficial cement cones, meaning that only a fraction of the mouthparts is inserted into the host tissue and their proximal parts are fixed by the cement cone above the skin surface. An example of this is *Ixodes tasmani* (Fig. 4), whose females insert their long mouthparts only 130 µm into the host, whereas the cement cone supporting the rest of the mouthparts at the skin surface measures 340 µm (Moorhouse, 1969).

Although there is some uniformity in cement shapes within tick genera (Fig. 4), the association of a particular cement configuration with a specific genus is not always possible (Mueller‐Doblies & Wikel, 2005). Chinery (1973) found differences between the cement cones of *Haemaphysalis spinigera* and the *Haemaphysalis* species described by Moorhouse (1969). Additionally, particular cement cone shapes may be influenced by the insertion angle of the mouthparts (Chinery, 1973).

## VIII. HISTOCHEMISTRY OF THE CEMENT

In different species, the cement substance can be either strongly eosinophilic (Saito & Ohara, 1961; Theis & Budwiser, 1974) or not eosinophilic (Cowdry & Danks, 1933). Moorhouse & Tatchell (1966) stated that the eosinophilic behaviour of the cement depends on the fixation and stain used, which may explain the reported variations.

Based on histochemical staining, the cement of *Rhipicephalus microplus* consists of protein (Bromophenol Blue) with additional lipid (Sudan Black B) in the core cement and carbohydrate‐containing (PAS) proteins in the cortical cement (Moorhouse & Tatchell, 1966). In addition to proteins, free lipid structures and carbohydrates are also found in cement cones from some, but not all, tick species examined to date (Moorhouse, 1969). Cement cones from the genera *Amblyomma* and *Bothriocroton* (formerly *Aponomma*) (Moorhouse, 1969) as well as *Haemaphysalis spinigera* (Chinery, 1973), *Hyalomma anatolicum* (Gill & Walker, 1988) and *Rhipicephalus appendiculatus* (Walker & Fletcher, 1986) seem to lack carbohydrates. As with the salivary granules mentioned above, PAS tests did not allow further differentiation of carbohydrates.

Lipids and/or carbohydrates are either found in distinct regions of a core and cortical cement or form a series of folded lamellae, each containing lipids and carbohydrates together with proteins, as described for *Dermacentor* species (Moorhouse, 1969). These patterns presumably develop from the sequence, dynamics and material properties of deposition.

Despite the longer activity of the cement‐producing salivary gland cells in males, at the histochemical level there are no differences between the cement produced by males and females of *Haemaphysalis spinigera* (Chinery, 1973).

Within cement, enzymes may be expected to be present as residuals from intracellular cement formation, the curing process or as stored catalytic material required for later cement degradation. A strong and homogeneous histochemical reaction for amino peptidase (leucyl naphthylamide method) was reported in the cement cones of *Hyalomma anatolicum* (Gill & Walker, 1988), *Rhipicephalus appendiculatus* (Walker & Fletcher, 1986) and in cement produced by larval *Rhipicephalus microplus* ticks (Schleger & Lincoln, 1976). Amino peptidases catalyse the cleavage of amino acids from the amino terminus of proteins or peptide substrates (Taylor, 1993). The function of amino peptidase in the largely proteinaceous cement material is unclear (Gill & Walker, 1988). Due to its presence throughout the cement cone, it is unlikely that this enzyme is involved only in modifications of fresh cement material. It may be a remnant of saliva portions used in the generation of the feeding pool. This might explain positive reactions for amino peptidase in areas of the host dermis at the attachment sites of *R. microplus* larvae (Schleger & Lincoln, 1976). Alternatively, this enzyme may assist in the detachment of the mouthparts from the cement after feeding, although there is no supporting evidence for this to date.

A weak reaction for acid phosphatase was identified with the Azo dye/lead method in cement cones of *Hyalomma anatolicum* (Gill & Walker, 1988), leading to suggestions that this enzyme has a gluing function in the cone (Walker *et al*., 1985; Gill & Walker, 1988), but this was not further investigated. Activity of acid phosphatase during the curing process would contribute to protein aggregation or polymerization through an increase in negative charge.

Histochemical staining (fast red salt B, diazotization) was also applied to test cement cones of *R. microplus* (Moorhouse & Tatchell, 1966) and *R. appendiculatus* (Walker & Fletcher, 1986) for phenolic groups. Phenol oxidase was identified in the salivary glands of the same tick species (Binnington, 1978; Walker *et al*., 1985), leading to suggestions that it was involved in a hardening process similar to insect cuticular tanning. However, positive phenol reactions were restricted to the cortical cement of *R. microplus* (Moorhouse & Tatchell, 1966), meaning that either tanning is only one of several curing mechanisms, or that phenols have other functions in tick cement.

As discussed above for intracellular granules, it is possible that the observed enzymatic reactions are not involved in cement formation, but relate to other functions of the saliva. Particularly surprising is the strong positive reaction for aminopeptidase; high concentrations of this enzyme would appear counterproductive in the protein‐rich cement, raising the question of how the cement cone is protected from such activities.

## IX. THE CURING PROCESS OF CEMENT

The curing of adhesives can take place by different mechanisms: hardening by loss of water or another solvent, cooling, or chemical reactions (Petrie, 2007). The latter include: (*i*) formation of helices from polysaccharides or proteins after cooling, supported by formation of hydrogen bonds and hydrophobic interactions (e.g. agar, gelatin); (*ii*) formation of ionic bonds between polysaccharides further stabilized by cations like Ca^2+^ (e.g. pectin); (*iii*) crosslinking of proteins by disulfide bonds, hydrophobic bonds (e.g. barnacle cement) or DOPA‐rich regions (e.g. mussel byssal plaques) (Smith, 2002).

It remains unclear which chemical process is responsible for the solidification of tick cement. It was suggested to involve a quinone tanning process (Moorhouse & Tatchell, 1966) or a reaction similar to the coagulation of haemolymph (Kemp *et al*., 1982). Quinone tanning is the chemical crosslinking of proteins by quinones of different structure. It is known to be involved in the sclerotization of insect cuticles and the formation of water‐resistant adhesives in marine mussels (Hong, Lee & Lee, 2014). Insect sclerotization is caused by crosslinking of phenols or primary and secondary amines of cuticular proteins with N‐acetyl‐catecholamines. In mussel adhesives, the oxidation of DOPA induces DOPA–quinone linking which rapidly forms covalent bonds with basic amino acids such as lysine and histidine (Hong *et al*., 2014).

The presence of phenolic groups both in cement cones and salivary glands of *R. microplus* and *R. appendiculatus* could suggest that a hardening process, similar to insect cuticular tanning (Binnington, 1978) or quinone tanning of neighbouring sulphydryl groups (Moorhouse & Tatchell, 1966) takes place, as in the cuticular tanning of the mite *Acarus siro* Linnaeus, 1758 (Hughes, 1959). However, reported low levels of sulphur‐containing proteins in the cement of *R. microplus* makes the latter mechanism unlikely (Binnington & Kemp, 1980). Further, phenolic groups were localized to cortical cement of *R. microplus* (Moorhouse & Tatchell, 1966) so a tanning process cannot explain the solidification of the core cement. In *Hyalomma anatolicum*, tanning similar to *A. siro* is unlikely to occur because sulphydryl and disulfide groups were detected at only very low levels and DOPA oxidase activity was not present (Gill & Walker, 1988).

Coagulation of the haemolymph was suggested to explain rapid solidification of core cement (Kemp *et al*., 1982). However, preliminary studies in *R. microplus* revealed differences in the amino acid composition of coagulated haemolymph and cement, and it was questioned whether solidified haemolymph has the necessary insolubility required for cement to persist over days in host tissue (Kemp *et al*., 1982).

Two chitinase‐like proteins [glycoside hydrolase 18 (GH‐18) family] were identified in the salivary glands of *Amblyomma americanum*. Their inactivation (silencing by RNA interference) impaired cement deposition leading to a significant decrease in attachment; treated ticks could be detached with a light touch. Furthermore, their feeding sites often showed extensive bleeding (Kim *et al*., 2014). Both proteins lack putative chitin‐binding domains and are also expressed in other parts of the tick body (Kim *et al*., 2014). Recently, two chitinases have been identified from the saliva of *Ixodes scapularis* Say, 1821, one of them inactive and with 64–65% similarity to the chitinase‐like proteins from *Amblyomma americanum*. However, the role of these proteins was not investigated (Kim *et al*., 2016).

Differences in curing and grade of solubility between the core and cortical cement may mean that several different processes contribute to the solidification of cement.

## X. TICK DETACHMENT

The mechanism behind tick detachment from the cement when leaving the host is not known. *Dermacentor andersoni* are able to separate within 1–2 min from the cement. Forced removal of tick and cement from ears of mice (Gregson, 1960) showed that the mechanical retraction of the chelicerae might be sufficient to achieve rapid detachment, but the secretion of a saliva component that dissolves the cement before detaching was also discussed (Kemp *et al*., 1982; Sauer *et al*., 1995). Bullard *et al*. (2016) reported that microlitre amounts of saliva can dissolve tick cement rapidly. Due to the high protein content of the cement, it might be expected that a protease is involved.

## XI. BIOCHEMISTRY OF CEMENT

Based on biochemical analyses of cement, the main amino acids present are leucine, serine, tyrosine and glycine, but only low concentrations of histidine and methionine are present (Kemp *et al*., 1982). Due to the fact that the cement cannot be easily dissolved, protein identification is very difficult. The presence of various binding mechanisms may support its poor solubility (Moorhouse & Tatchell, 1966; Kemp *et al*., 1982). The best solubilization can be achieved by using hot bases or acids (Kemp *et al*., 1982), which, however, makes proteomic analysis almost impossible because of hydrolysis. Several proteins are known to be present in tick cement: examples are a 94 kDa protein detected from various tick species, a 20 kDa protein of *Amblyomma americanum* and a 15 kDa protein of *Rhipicephalus appendiculatus*, named 64P (Brown, Shapiro & Askenase, 1984; Shapiro, Voigt & Fujisaki, 1986; Shapiro, Voigt & Ellis, 1989; Havlikova *et al*., 2009).

The introduction of artificial feeding systems on silicone membranes allowed the isolation of cement cones of *Amblyomma americanum* free from host skin tissue for comparison with cement from *in vivo*‐fed ticks of this species (Bullard *et al*., 2016). Cement originating from *in vitro* feeding allowed the identification of more proteins than cones harvested from *in vivo* systems. In cement from membrane‐fed ticks, 26 proteins were identified with liquid chromatography tandem mass spectrometry (LC‐MS/MS), including some intracellular proteins, glycine‐rich proteins (GRPs; containing more than 20% glycine), serine protease inhibitors, metalloproteases and unclassified proteins. Bullard *et al*. (2016) hypothesised that GRPs may stabilize and strengthen the cement cone and contribute to its insolubility.

The metalloproteases identified in cement may prevent blood clotting during feeding. Blood clotting may also be reduced by the inhibition of thrombin by protease inhibitors. The function of many of the identified proteins remains unknown, especially as these proteins are not yet characterized. Moreover, the low levels of arginine and lysine residues present in larger peptides after a typical enzymatic approach using trypsin for protein identification makes detection and sequence determination by MS and MS/MS difficult (Tan *et al*., 2015).

Among amino acids, glycine is the best understood to date. Glycine was also reported to be the most abundant amino acid in the cement of *Rhipicephalus microplus* (Kemp *et al*., 1982), and high concentrations of this amino acid are also known from other biological adhesives. High glycine contents were found in isolated saliva proteins of *Haemaphysalis longicornis* Neumann, 1901 and *Rhipicephalus appendiculatus* with a proven or putative role in cement formation (Mulenga *et al*., 1999; Bishop *et al*., 2002; Trimnell *et al*., 2005; Zhou *et al*., 2006). To date, only one GRP has been isolated from *in vitro* cement cones of *Amblyomma americanum* (Bullard *et al*., 2016). This might be because only a certain cement fraction was investigated (Bullard *et al*., 2016). Current opinions on the function of GRPs in the cement of ticks are summarized in Table 6.

**Table 6:** Suggested functions for glycine‐rich proteins (GRPs) in tick cement cones and similarities to other proteins

Functions/properties of GRPs	References
Mimic components of the vertebrate host to use host‐derived enzymes for the cement hardening process to inhibit rejection by the host to facilitate the binding between cement and host tissue	Bishop *et al*. (2002), Havlikova *et al*. (2009) and Trimnell *et al*. (2002)
Similarity to extracellular matrix proteins (e.g. keratin, collagen, loricrin)	Bishop *et al*. (2002), Francischetti *et al*. (2009), Havlikova *et al*. (2009) and Trimnell *et al*. (2002)
Similarity to spider silk proteins	Francischetti *et al*. (2009) and Maruyama *et al*. (2010)
Similarity to peptides with antimicrobial activity from *Caenorhabditis elegans* Maupas, 1900	Francischetti *et al*. (2009)

If GRPs are involved in cement production, then it might be the case that species with short mouthparts produce more GRPs than species with long mouthparts, because they depend on cement as part of their anchoring mechanism. Similarly, one‐host ticks, which spend most of their life attached to the host, might produce larger quantities of these proteins. The identification of nucleotide sequences which encode potential GRPs from cDNA libraries of three different tick species [*Amblyomma cajennense* (Fabricius, 1787), *Rhipicephalus microplus*, *R. sanguineus*] seems to confirm these suggestions (Maruyama *et al*., 2010). However, GRPs may have other functions: they are also found in antimicrobial substances and are related to immune evasion (Francischetti *et al*., 2009; Havlikova *et al*., 2009). Proteins of this class are also found in the family Argasidae (Maruyama *et al*., 2010), which do not produce cement. Additionally, some GRPs in tick saliva are shorter than proteins usually involved in animal adhesives such as spider silk (Bullard *et al*., 2016).

In the saliva of *Ixodes scapularis*, 17 GRPs have been identified that were present in the early phase of attachment and also at the end of feeding and after detachment. It was suggested that the latter might be the result of degenerating processes of the salivary glands at that point (Kim *et al*., 2016). Further analysis of tick cement composition used Fourier transform infrared spectroscopy attenuated total reflectance (FTIR‐ATR) to obtain information about the secondary structures of proteins at the surface of the cement cone (Bullard *et al*., 2016). They observed significant differences between *in vivo* and *in vitro* collected cement cones. Both contained β‐sheet structures and usually β‐turns. Cement cones from *in vivo*‐fed ticks additionally contained helical protein conformations (perhaps due to proteins synthesized by the host skin) but most of the structures were β‐sheets. In comparison, the cement cone of *in vitro*‐fed ticks exhibited proteins with mainly random coil structures, seen in cement after 72 h of feeding, with β‐sheets and β‐turns appearing later, after 5–7 days. These findings indicate that a conformational shift is involved in the curing/hardening process.

## XII. COMPARISON OF CEMENT WITH OTHER BIOLOGICAL ADHESIVES

Other animals also produce cement‐like adhesives for attachment. The barnacle *Semibalanus balanoides* (Linnaeus, 1767) (formerly *Balanus balanoides*), uses a special glue to adhere to surfaces under water. The cypris larvae (the final larval stage of barnacles) uses its attachment organ to anchor to the substratum while exploring it for permanent attachment at a later stage (Nott, 1969). Its glue production is located in round or oval unicellular gland cells, with a diameter of up to 200 µm (Fyhn & Costlow, 1976). During exploration of new habitats, proteinaceous ‘footprints’ of antennular secretions from previously arrived larvae stimulate settlement and may enhance the attractiveness of a surface, resulting in gregarious settlement even if no conspecific adult is present (Yule & Walker, 1984; Clare, Freet & McClary, 1994; Matsumura *et al*., 1998). When the final site has been selected, permanent glue, a clear non‐viscous fluid, is produced, which increases in viscosity and cures over the course of minutes to hours forming an approximately 5 µm layer of a multiprotein complex called ‘cement’ (Saroyan, Lindner & Dooley, 1970; Cheung, Ruggieri & Nigrelli, 1977; Kamino, 2008). The tensile strength of the cement of adult barnacles was shown to be stronger than mussel or limpet adhesives, but weaker than commercial dental adhesives (Yule & Walker, 1984; Nakajima *et al*., 1995). The cement contains more than 90% protein (Walker, 1972; Kamino, Odo & Maruyama, 1996). However, DOPA – a common component in the foot proteins of the mussel – is not known from barnacle cement (Kamino *et al*., 1996). Total protein hydrolysis showed that serine, threonine, alanine, glycine and proline are the most abundant amino acids in barnacle cement, but interspecific differences were found (Walker, 1972; Kamino *et al*., 1996; Kamino, 2008). Lipids and carbohydrates are present in trace amounts, and silicon, calcium, aluminium and iron are the major inorganic residues (Walker, 1972). Several soluble proteins (hydrophobic, six amino acid biased, charged amino acid rich) were identified, but their functions are not fully understood. Hypotheses about underwater attachment functions including priming, spreading and condensation and possible protection against bacterial degradation by lytic activity have been suggested (Kamino, 2006). Accurate protein analysis of the barnacle cement is again prevented by its solubilization. High solubilization (>90% of the cement of *Megabalanus rosa* Pilsbry, 1916) was enabled using guanidine hydrochloride (GdnHCl), a strong chaotrop and one of the strongest denaturants, under reducing conditions using dithiothreitol (DTT) (Kamino *et al*., 2000). This non‐proteolytic solubilization method revealed the importance of disulfide bonds for the stability of the cement and that certain bulk proteins are responsible for cement firmness (Kamino, 2008).

Like barnacles, the *Mytilus* genus of mussels also attaches to various substrata (e.g. rocks, wood, seaweed, other animals or ship hulls). This is mediated through a sectorial organ called the byssus, which can be divided into three main parts: the proximal part (root) and the distal part (stem) of the collagenous threads, and the attachment plaque (Brown, 1952). The adhesive proteins are produced within the foot organ, accumulated and then secreted into the sea water, where they produce byssal threads and the adhesive plaque through a curing process within minutes (Silverman & Roberto, 2007; Bandara, Zeng & Wu, 2013). Qin *et al*. (2016) identified 48 byssal proteins including collagens, protease inhibitors, enzymes, and other unknown proteins. Of these 48 proteins, 11 were exclusively from the plaque and therefore directly involved in the adhesion process. Particularly interesting was the identification of plaque enzymes including tyrosinase, superoxide dismutase, amino oxidase, glycosyl‐hydrolase and peroxidase. DOPA is a common post‐translationally modified tyrosine in mussels, and thus the presence of tyrosinase enzymes is expected: eight such enzymes were found, oxidizing tyrosine to DOPA and further DOPA to o‐quinone leading to tanning, which has been proposed to play a focal role in mussel adhesion and cohesion (Waite *et al*., 2005; Anderson *et al*., 2010; Niklisch & Waite, 2012). The identified peroxidase enzymes may also be part of this redox balance system. Other abundant amino acids are proline, glycine, tyrosine, lysine and asparagine (Gantayet, Rees & Sone, 2014). A thin cuticular layer (5–10 µm) coating the byssal threads protects the mechanical integrity of this fibrous structure. This coating has an unusual combination of high hardness and extensibility, attributed to the formation of protein–metal complexes between three DOPA molecules from the mussel foot protein 1 (mfp‐1) and one metal ion (Fe, V, Al) (Schmitt *et al*., 2015).

Another animal that secretes a proteinaceous glue is the sandcastle worm *Phragmatopoma californica* (Fewkes, 1889), a marine polychaete, which uses the adhesive to build a surrounding protecting tube. The cementing material, which adheres sand and shell fragments together, is produced in bilateral glands and secreted from a special organ (Jensen & Morse, 1988). The cement bonds and the whole tubular construction must be strong and stable to withstand the turbulent, high‐energy environment of the intertidal zone (Stewart *et al*., 2004). After secretion, the cement appears creamy white or light tan and turns reddish to dark brown as it ages (Jensen & Morse, 1988; Stewart *et al*., 2004). It sets in only 30 s, but takes several hours to cure into a solid foam (Stevens *et al*., 2007). The adhesive material is largely proteinaceous containing 59.5% of total amino acid residues as short‐chain amino acids (alanine, glycine, serine) and 2.6% DOPA which is thought to act as a cross‐linker, stabilizing (tanning) the cement (Jensen & Morse, 1988; Stewart *et al*., 2004; Stevens *et al*., 2007). In addition, the cement also contains considerable amounts of phosphorus, calcium, magnesium and sulfur (Stewart *et al*., 2004; Sun *et al*., 2007; Wang & Stewart, 2013). Several charged polyelectrolytic proteins are present mainly composed of glycine, lysine, tyrosine (mostly as DOPA) and (phospho)‐serine (Waite, Jensen & Morse, 1992; Zhao *et al*., 2005; Endrizzi & Stewart, 2009). Zhao *et al*. (2005) proposed a process of cement formation where some or all of the precursors are accumulated with Ca and Mg as multiphase coacervates. These coacervates release the glue, which gelates in the sea water because of the lower solubility of Ca/Mg phosphate, and irreversible cysteine–DOPA cross‐links are formed (dopaquinones). This cross‐linking is catalysed through monophenoloxidase and catechol oxidase activity of a tyrosinase, which is present in the secretory cells (Solomon, Sundaram & Machonkin, 1996; Klabunde *et al*., 1998; Wang & Stewart, 2013).

The sea star *Asterias rubens* Linnaeus, 1758 secretes an adhesive material used for dynamic attachment to withstand wave movement or to grip and pry open a mussel during feeding. Several studies (Hennebert, Wattiez & Flammang, 2011; Hennebert *et al*., 2012, 2015) investigated the proteome of the sea star and the composition of the temporary adhesive, which mainly consists of proteins and carbohydrates. Several proteins of the glue secreted from the tube feet could be identified, despite its low solubility. Again, electrostatic interactions (polar and hydrogen‐bonding of functional groups of glycan chains) and cross‐links between the proteins may be responsible for its cohesive and adhesive strength. In addition, mucin‐like proteins were identified which could be involved in the formation of a structural network through cross‐linking or oligomerization to other molecules. Furthermore, proteins similar to metalloendopeptidases were identified in the glue and may be involved in the detachment process by degrading adhesive proteins. Several lectins were used to recognize oligosaccharide motifs on tube foot sections and on whole footprints. For the latter concanavalin A (Con A), wheatgerm agglutinin (WGA), ricin (RCA) and *Dolichos biflorus* agglutinin (DBA) showed positive reactions. No detailed information regarding the molecular mechanisms underlying sea star adhesion is yet available.

Another echinoderm, the sea urchin, also secretes a proteinaceous and carbohydrate‐rich, reversible adhesive *via* its tube feet. Many proteins in the adhesive and non‐adhesive part of this secretion have been identified, but only some have been characterized further (Lebesgue *et al*., 2016). Lebesgue *et al*. (2016) proposed a molecular model for sea urchin reversible adhesion with putative functions of the identified proteins. Nectin is one of the main adhesive proteins in addition to cohesive proteins, proteoglycans, curing and antimicrobial proteins. Again proteases and glycosylases may be the main de‐adhesive secretion components.

The biochemical analysis of tick cement is still at an early stage. There are no detailed data regarding the metabolites, proteins, lipids and carbohydrates present in tick cement. Most proteins identified to date are from the salivary glands and their presence in the cement is speculative. Moreover, the adhesive mechanisms on which the secreted cement material is based remain unknown. Detachment mechanisms after the blood meal also remain theoretical (Gregson, 1960; Kemp *et al*., 1982; Sauer *et al*., 1995). However, there are several similarities between tick cement and bioadhesives from other animals. In all cases, the poor solubility makes analysis challenging. Tick and barnacle cements are mainly proteinaceous (above 80 and 90%, respectively), but protein identification is difficult, requiring a mild solubilization strategy. However, it is known that the amino acid composition of tick cement is similar to other adhesives. Glycine, serine, proline, alanine and tyrosine are abundant in the cement in all cases. A high content of short‐chain amino acids may be correlated with cross‐linking or hardening processes of the cement giving it high stability. Furthermore, the presence of DOPA is essential for the gluing function of the adhesives of mussels and sandcastle worms, but it has not yet been proven in tick or barnacle cement. The cement of both the sandcastle worm and the tick contains phenolic groups and a phenol oxidase. Tyrosinase activity is important in the formation of cross‐links and phenolic groups could be involved in the hardening process of the adhesive.

## XIII. TICK SALIVA AS A SOURCE OF TOXINS, TICK‐BORNE PATHOGENS AND NEW THERAPEUTICS

The saliva produced by ticks is extremely complex and contains several hundred proteins (Brossard & Wikel, 2008) and other non‐proteinaceous biomolecules (Oliveira *et al*., 2011) that are secreted into the host. Some of these are irritating and skin‐sensitizing substances or toxins. For instance, proteinases in tick saliva may act as digestive enzymes (Tirloni *et al*., 2015). Proteins of the lipocalin family are found in Argasidae and Ixodidae and are thought to modulate inflammation processes; some lipocalins are responsible for ‘sand tampan toxicoses’ resulting from bites of *Ornithodoros savignyi* (Audouin, 1826). In addition, a number of proteins (e.g. cystatin, kunitz, metalloprotease) in tick saliva are known as components of toxic secretions from venomous animals (Estrada‐Peña & Mans, 2014).

Tick saliva also constitutes the main route for the transmission of tick‐borne pathogens which include viruses, bacteria and parasites (e.g. Tick‐borne encephalitis virus, Crimean‐Congo haemorrhagic fever virus, *Borrelia burgdorferi sensu lato*, *Rickettsia rickettsii*, *Francisella tularensis*, *Ehrlichia chaffeensis*, *Coxiella burnetii* and *Babesia microti*). Once acquired from a tick vector, pathogens can be transmitted from larvae to nymphs or nymphs to adults by transstadial transmission and/or to the next generation by transovarial transmission (Macaluso & Paddock, 2014; Nuttall, 2014). The immunomodulatory activity of saliva which allows the tick to feed successfully also leads to a phenomenon called saliva‐assisted transmission (Labuda & Nuttall, 2008). The local conditions at the tick feeding sites, comparable to protected niches within the host body (Nuttall & Labuda, 2008), facilitate the survival of pathogens and transmission to previously uninfected ticks (Randolph, 2011).

Compounds of tick saliva have been considered as medical therapeutics in recent years. Investigations have included studies on anti‐tumoral effects (Chudzinski‐Tavassi *et al*., 2010; Abreu *et al*., 2014; Sousa *et al*., 2015), applications against inflammatory diseases like myasthenia gravis (Hepburn *et al*., 2007), new anticoagulants (Koh *et al*., 2011) and the treatment of asthma (Horka *et al*., 2012).

## XIV. POTENTIAL APPLICATIONS OF TICK CEMENT

The natural adhesive and sealing functions of tick cement in vertebrate skin tissue suggest potential applications in medicine, in particular as a tissue glue or sealant. However, such tick cement‐based glues will depend on as yet unknown properties of the adhesive molecules, which need to be identified and carefully studied.

There is a high demand for adhesives in medicine, as established tissue glues contain either toxic substances (cyanoacrylates, glutaraldehyde) or have weak bonding forces (fibrin glues) (Fuerst & Banerjee, 2005; Schneider, 2009; Zhang *et al*., 2014b). Furthermore, existing tissue glues do not cover all possible fields of application: sutures, staples, screws and plates could be replaced by glues in some applications, if they can provide particular properties (Duarte *et al*., 2012). Additionally, the trend towards minimally invasive surgical methods (laparoscopic, endoscopic, and robotic techniques) further increases the requirement for adhesives, as suturing and similar procedures are difficult through small incisions (Spotnitz & Burks, 2008; Schneider, 2009).

Tick cement may be an exciting answer that opens up new fields of application. Despite the fact that the properties of the adhesive molecules in tick cement remain unknown to date, observations suggest that tick cement contains two types of adhesives that differ functionally in curing time: a rapidly curing (core cement) and a slower hardening (cortical cement) fraction (see Section IV). Fast‐curing adhesives might be used to close wounds or to stop bleeding, for example in liver lacerations. The cortical cement might have applications where longer time frames are required for tissue manipulation such as during reconstruction of complex fractures.

Tick cement has a viscous consistency. If this is also true for its adhesive component, it could allow fixing of small bone fragments too small for screw fixation and currently stabilized by sutures or pins. Since autologous bone material is still considered to be the best material for bone regeneration, the preservation of small bone granules is of considerable importance. Cement might also be used for larger unloaded bone fragments such as the skullcap to avoid screwing and metal plate fixation. Gluing of tendons and ligaments without suturing or screwing would improve refixation, but is especially challenging as exceptional tensile strength is required. Fluent formulations could be applied to large surfaces in skin regeneration or hernia net fixation and in a similar method to fibrin spray applications (Sawamura *et al*., 1999).

The likely presence of both a rapid‐ and a slow‐hardening fraction in cement makes possible the sequential use of two components: prefixation with a faster curing glue and final stabilization with the slow‐curing type. A mixture could result in a glue with hybrid properties, giving more application flexibility. In Section X we considered how ticks appear able to detach from their hosts by dissolving the cement with their saliva. A putative cement‐dissolving portion of saliva enzymes could thus be considered as a component for a reversible tissue glue. Today, no enzyme‐based, reversible glue is available to our knowledge, but this novel concept would facilitate repositioning of tissue fragments in complicated surgery or their removal at a later time point.

Recently it has been shown that it is advantageous to glue intravenous catheters to the patient's skin to avoid dislodgement, micromotion leading to vein irritation or bacterial invasion from the skin (Bugden *et al*., 2016). This could be another application of cement‐based adhesives. Similarly, the fixation of cannulas which must remain in place for longer time periods, e.g. in intracerebral brain infusion in which the curved skull shape makes it difficult to fix cannulas properly (Sike *et al*., 2017) might be possible.

Besides medical applications, the cosmetic industry is always exploring new concepts and components for their products. Among the proteins applied in cosmetics are collagen, elastin and the silk protein fibroin, which all have a high content of the amino acid glycine (Secchi, 2008). Tick cement contains high levels of GRPs (see Table 6) making it a potential new constituent for cosmetics.

Application of biological compounds to industrial products requires a long process of isolation, identification and manipulation of ingredients to achieve optimal results in durability, applicability and biocompatibility. As such, biotechnological processing and chemical modification very often improve the final products. Biochemical techniques may allow the introduction of chemical modifications to tick‐cement constituents, to insert beneficial protein sequences into sequences of tick‐cement proteins or to combine other components to form new adhesives. Such engineering has already been successfully used for DOPA, an important component in mussel adhesion (see Section XII). Today, DOPA is copolymerized with vinyl monomers to form dopamine‐co‐acrylate, a hyperbranched polymer with a fourfold increase in wet adhesion strength compared to commercial fibrin sealants (Zhang *et al*., 2014b). In another study, a new DOPA‐based adhesive for rapid emergency re‐entry after sternotomies was designed by using initially stable, but gradually hardening substances (Zhang *et al*., 2014a). DOPA was also combined with polyethylene glycol to result in a photopolymerizable gel with sufficient strength for applications in skin and heart in *ex vivo* animal models (Zhang *et al*., 2015).

We conclude that tick cement has much potential as a template for a biomimetic tissue adhesive and is an exciting resource for future developments in biomedical engineering.

## XV. CONCLUSIONS

(1) The production of cement appears to be an exclusive feature of ixodid ticks and is related to the high complexity of the salivary glands in this tick family. Relatively few species of this tick family feed without the production of the adhesive, emphasising the importance of this substance to the enormous success of the Ixodidae as long‐term blood feeders. Besides its more obvious functions as an adhesive and sealant, cement production might also allow the tick to avoid detection by the host and its defence systems. The reduction of tissue damage, the exclusion of bacteria from the host skin surface and the nearly complete isolation of the mouthparts from surrounding tissues are all facilitated by superficial cement deposition.

(2) As the largest family of ticks, the Ixodidae offer a number of opportunities for comparative research on cement. If differences in cement exist among species they will most likely be detected by studies on ticks with a narrow host spectrum or extreme host specificity. Such species could act as model organisms for adaptations of the adhesive to particular vertebrate groups or species. Comparative research on host‐specific ticks may also help to identify components that are evolutionarily conserved and might therefore be critical for adhesiveness. Such studies will also contribute to our understanding of rare host specialisations, like feeding on amphibians. Subsequent identification of genes coding for cement components could also result in suitable alternative markers for molecular‐based phylogenetic studies and tick systematics and consequently improve our knowledge on the phylogenetic relationships among Ixodidae.

(3) The artificial feeding of ticks on silicone membranes appears to be a suitable tool for studies on cement material, offering the possibility of uncompromised samples (free of skin tissue). Artificial feeding allows access to the adhesive material at virtually any time during feeding, something which is not possible *in vivo*. In this way the progress of cement deposition can be monitored. The development and standardization of mechanized feeding systems, allowing automatic and simultaneous artificial feeding of large numbers of ticks in order to yield quantities of cement material for analyses would be desirable. To obtain samples of high purity, a further improvement of the method might be the introduction of artificial feeding media to exclude host compounds derived from blood.

(4) The medical importance of ticks is generally associated with their vector role in disease transmission, but there are increasing examples that some tick products might be beneficial to man. Some salivary gland products are promising starting compounds for new therapeutics and, similarly, tick attachment cement might become a rich source of innovative products in medicine. In contrast to many other adhesives considered for future medical applications, tick cement is used in nature in a similar way, suggesting that this adhesive might be especially suitable for the development of new medical adhesives. Due to the natural function of the attachment cement in adhesion to and sealing of vertebrate skin, it might be especially promising as a tissue adhesive or sealant. Apart from the gluing function, there are two other characteristics of interest for medical applications: (*i*) the observed similarities of cement components to proteins found in vertebrate tissue might suggest good integration of its components and tissue compatibility; (*ii*) its possible antimicrobial activity.

(5) Research on tick cement is still in its infancy and currently little is known about the compounds involved in the formation and solidification of cement. Most information available comes from only a small number of tick species and much remains speculative. By contrast, more molecular biological information is available for potential compounds in the saliva of ticks. These components are probably also present in the cement, either as components or residuals. Besides the identification of the chemical components involved in the gluing function and the processes responsible for solidification, studies on the mechanical properties (e.g. elasticity, hardness, tensile strength) and surface interactions of cement are also lacking. Such studies will be of major importance for possible applications of cement‐based adhesives. Concerted interdisciplinary efforts and highly sophisticated methodologies are needed to reach a better understanding of cement and its underlying adhesion mechanisms.

## XVI. ACKNOWLEDGEMENTS

This study was funded by the Austrian Science Fund (FWF, Grant no. P 28962) and supported by the European Cooperation in Science and Technology (COST Action TD0906 and CA15216). We thank Daniela Gruber, Core Facility Cell Imaging and Ultrastructure Research (CIUS) and Thomas Schwaha, Integrative Zoology, both at the University of Vienna for access to electron and light microscopic facilities. Further, we thank Christoph Hörweg, Natural History Museum Vienna for providing specialized literature. The comments and suggestions of two anonymous reviewers and the Editor‐in‐Chief are greatly acknowledged.
